# Edaravone Dexborneol Treatment Attenuates Neuronal Apoptosis and Improves Neurological Function by Suppressing 4-HNE-Associated Oxidative Stress After Subarachnoid Hemorrhage

**DOI:** 10.3389/fphar.2022.848529

**Published:** 2022-04-21

**Authors:** Qian Chen, Yichen Cai, Xiaoyu Zhu, Jing Wang, Feng Gao, Mingfeng Yang, Leilei Mao, Zongyong Zhang, Baoliang Sun

**Affiliations:** The Second Affiliated Hospital, Brain Science Institute, School of Basic Medical Sciences of Shandong First Medical University and Shandong Academy of Medical Sciences, Taian, China

**Keywords:** oxidative stress, subarachnoid hemorrhage, edaravone dexborneol, 4-HNE, edaravone

## Abstract

Edaravone dexborneol is a novel neuroprotective drug that comprises edaravone and (+)-borneol in a 4:1 ratio. Phase II and III studies have demonstrated that Chinese patients treated with edaravone dexborneol within 48 h of AIS onset have better functional outcomes than those treated with edaravone alone. However, the effect of edaravone dexborneol on subarachnoid hemorrhage (SAH) has not yet been elucidated. This study aimed to investigate the therapeutic effects of edaravone dexborneol on SAH-induced brain injury and long-term behavioral deficits and to explore the possible mechanisms. The experimental rat SAH model was induced by an intraluminal puncture of the left middle cerebral artery (MCA). Edaravone dexborneol or edaravone at a clinical dose was infused into the tail vein for 3 days post-SAH surgery. Behavioral outcomes were assessed by a modified Garcia scoring system and rotarod, foot-fault, and corner tests. Immunofluorescence, Western blot, and ELISA methods were used to evaluate neuronal damage and oxidative stress. Our results showed that a post-SAH therapeutic regimen with edaravone dexborneol helped improve neurological function up to 21 days after SAH surgery and demonstrated a greater beneficial effect than edaravone alone, accompanied by an obvious inhibition of neuronal apoptosis in the CA1 hippocampus and basal cortex regions. Mechanistically, edaravone dexborneol not only suppressed the lipid peroxidation product malondialdehyde (MDA) but also improved the total antioxidant capability (TAC) 3 days after SAH. Notably, edaravone dexborneol treatment significantly inhibited the expression of another lipid peroxidation product, 4-hydroxynonenal (4-HNE), in the CA1 hippocampus and basal cortex, which are vital participants in the process of neuronal oxidative damage and death after SAH because of their acute cytotoxicity. Together, our results demonstrate that edaravone dexborneol confers neuroprotection and stabilizes long-term behavioral ability after SAH injury, possibly by suppressing 4-HNE-associated oxidative stress. These results may help develop new clinical strategies for SAH treatment.

## Introduction

Subarachnoid hemorrhage (SAH) is considered an uncommon and severe subtype of stroke, which accounts for only 5% of strokes, but occurs at a fairly young age (van Gijn et al., 2007; Macdonald and Schweizer, 2017). Moreover, the high disability and fatality rates of patients after SAH result in huge spiritual and financial burdens for the patients and their families (Macdonald and Schweizer, 2017). As one of the significant pathological mechanisms of SAH-induced brain injury, oxidative stress has been shown to induce neuronal apoptosis and tissue necrosis by producing excessive reactive oxygen species (ROS), MDA, 4-HNE, and so on (Yang et al., 2017; Liu et al., 2020; Wu et al., 2021). When oxidative stress occurs, 4-HNE is a vital participant in the process of DNA damage (Liu et al., 2020). Meanwhile, MDA is regarded as a key marker of oxidation in the cell membrane (Tsikas, 2017).

As a potent free radical scavenger, edaravone has been shown to protect ischemic neurons after stroke via its antioxidant property, including suppression of lipid peroxidation and oxidant-induced DNA damage (Kikuchi et al., 2013; Wu et al., 2014a). In addition, edaravone can efficiently diminish apoptosis of neurons and thus prevent the neurologic impairment after SAH (Munakata et al., 2011). At present, edaravone is widely used in the clinic as a neuroprotective agent. Notably, a novel neuroprotective agent called edaravone dexborneol, which comprises (+)-borneol and edaravone in a ratio of 1:4, has been recently synthetized in China (Xu et al., 2019). A phase III clinical trial demonstrated that Chinese patients treated with edaravone dexborneol showed better functional outcomes within 48 h of AIS onset than those treated with edaravone alone (Xu et al., 2021). Edaravone dexborneol is presumed to protect against AIS by multifunctional cytoprotective pathways, including oxidative, inflammatory, excitotoxic, and apoptotic insults (Wu et al., 2014b). We speculated that edaravone dexborneol treatment might attenuate SAH-induced brain injury by suppressing oxidative stress. Nevertheless, the therapeutic effects of edaravone dexborneol on SAH injury have not been evaluated.

In this study, we evaluated the protective effect of edaravone dexborneol on a rat model of SAH and explored the potential underlying mechanism. Our results indicated that compared with edaravone, edaravone dexborneol treatment conferred better protection against neurological deficits after SAH and that this effect was accompanied by the inhibition of neuronal apoptosis in the CA1 hippocampus and basal cortex. Furthermore, edaravone dexborneol treatment not only suppressed the lipid peroxidation product malondialdehyde (MDA) but also improved the total antioxidant capability (TAC method) 3 days after SAH. Notably, another lipid peroxidation product, 4-HNE, was also obviously inhibited by edaravone dexborneol in the CA1 hippocampus and basal cortex, which are vital participants in the process of neuronal oxidative damage and death after SAH because of their acute cytotoxicity. Our findings suggest that edaravone dexborneol treatment is a novel and clinically feasible therapy to protect the brain against SAH injury.

## Materials and Methods

### Animals and Experimental Design

Male Sprague–Dawley (SD) rats (14 weeks old, 280–300 g) were purchased from the Pengyue Laboratory Animal Center (Jinan, China). All animal experiments were approved by the Institutional Animal Care and Use Committee of Shandong First Medical University (Approval No: W202103030172) and conducted in accordance with the National Institutes of Health (NIH) Guide for the Care and Use of Laboratory Animals. All animals were housed in a temperature- and humidity-controlled 12-h light/dark cycle with *ad libitum* access to food and water.

**FIGURE 1:**
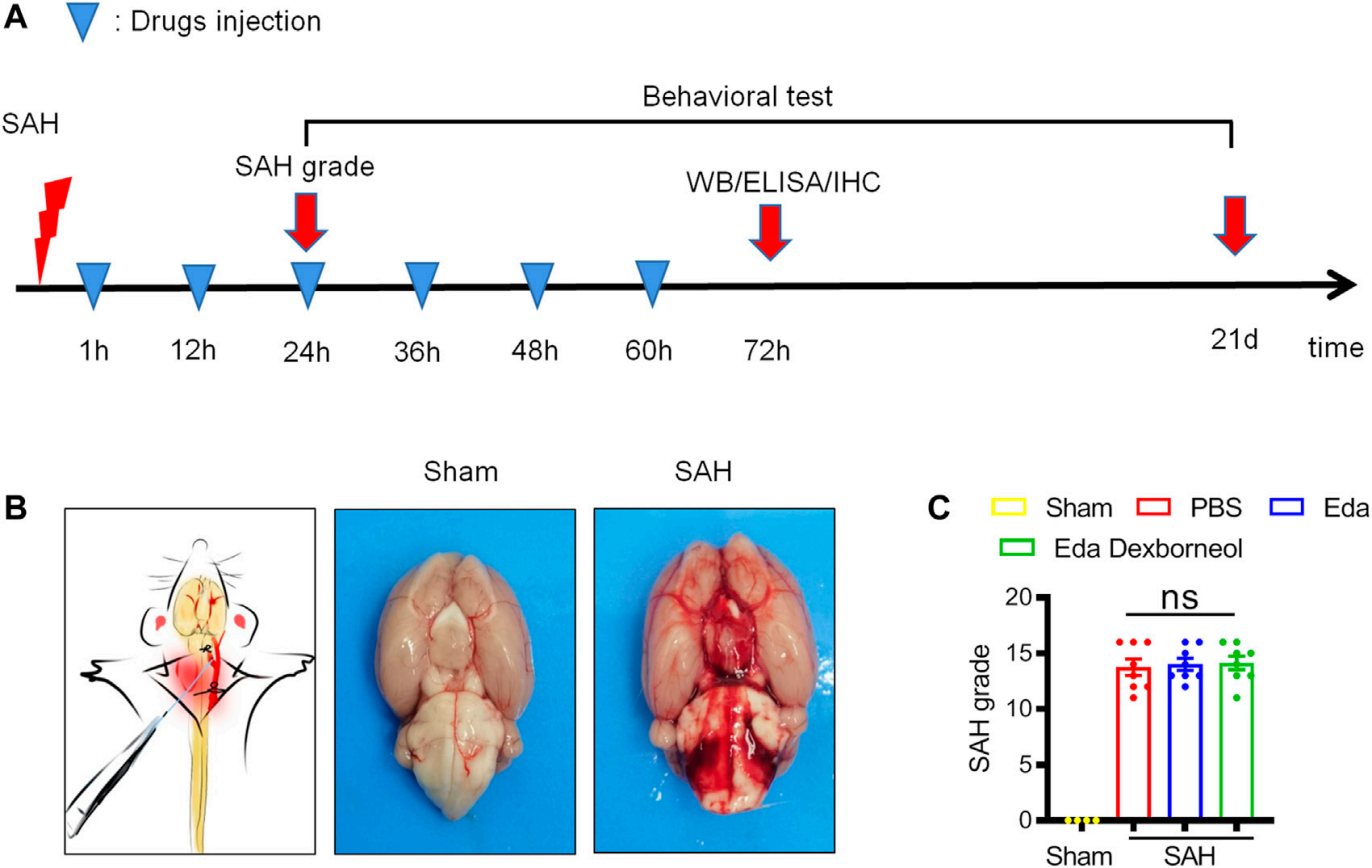
Establishment of a stable SAH rat model. **(A)** Diagramshowing the time points for drug injection and outcome evaluation. **(B)** Schematic representation of the SAH model (left) and representative images of rat brains in the sham/SAH group. **(C)** SAH grading scores of the four groups were assessed after 24 h. *n* = 4/group. Data are given as mean ± SE. ns: no significant difference; Eda: edaravone; Eda dexborneol: edaravone dexborneol.

All experiments were performed following the experimental design (Figure 1A). Rats were randomly assigned to experimental groups. Edaravone dexborneol, edaravone, or the same volume PBS was administered by tail vein injection for 3 days post-SAH surgery, and the specified dosage (edaravone dexborneol: 3.75 mg/kg, edaravone: 3 mg/kg, twice a day) was the determined reference to the clinical dose (Xu et al., 2021). All experiments were performed by an investigator blinded to experimental group assignments.

### Rat Subarachnoid Hemorrhage Model

The experimental SAH model was induced by intraluminal puncture of the left middle cerebral artery (MCA) as previously (Sugawara et al., 2008; Dong et al., 2021). In brief, rats were deeply anesthetized with 6% isoflurane and maintained with 2.5% isoflurane in a 30% O2/70% N2O mixture via a facemask by a rodent ventilator (RWD, China). In brief, an incision in midline neck skin was made, and then the left common, external, and internal carotid arteries were isolated. A blunted nylon (4-0) suture was inserted into the left internal carotid artery via the left external carotid artery and then moved toward the puncture of the left middle cerebral artery. The incoming length of the nylon suture was approximately 18–20 mm. Subsequently, the suture was withdrawn to enable reperfusion. Sham-operated animals underwent the same anesthesia and surgical procedures but were not subjected to intracranial endovascular perforation.

The success of the SAH models was evaluated by the SAH grade scoring system (Sugawara et al., 2008). The SAH grade score system includes two approaches: the bleed grade score system and the modified Garcia scoring system. The bleed grade score system (score <8 was excluded from the study) was used for animals that were killed 24 h or 3 d after SAH, and the modified Garcia scoring system (score >14 on day 1 after SAH was excluded from the study) was used 1 day after surgery for animals that needed to survive for a long time. The mortality rate for the SAH animals was approximately 5.4% (six of 113 total rats died after SAH surgery), and the success rate was approximately 91.6% (9 excluded of 107 rats).

### Behavioral Tests

Behavioral tests were performed in a blinded manner to evaluate the sensorimotor function of the rats. The modified Garcia scoring system was performed as described previously (2) and used to evaluate the comprehensive neurological function after SAH. In brief, the modified Garcia scoring system includes six items (3 points/item): body sensations, spontaneous activity of the extremities, motor ability, climbing ability, movement and strength of the forelimbs, and responses to stimulating whiskers.

The rotarod test was performed to evaluate the balance and sensorimotor coordination (Mao et al., 2020). After the animals were placed on the rods, the rods began to accelerate to 40 r/min within 30 s, and this speed was maintained until 300 s. The duration on the rods was recorded. The foot-fault test was related to deficits in motor control. The rats were placed on a stainless steel grid floor and allowed to move on it for 1 min during each trial. A foot fault was recorded when a paw slipped. The percentages of foot faults were calculated by errors versus total steps made by the contralateral limbs. The corner test was used to assess asymmetric behavior after SAH (Lei et al., 2020; Mao et al., 2020). The rats were placed between two boards angled at 30°. Normal rats randomly turned back without a preference for direction, but rats with focalized sensorimotor dysfunction showed a preference to turn toward the non-impaired side. We repeated the corner test ten times and recorded the number of right or left turns.

### Immunofluorescence Staining

Three days after SAH, the animals were euthanized and perfused with cold saline, followed by PBS containing 4% paraformaldehyde. Their brains were removed and fixed in 4% paraformaldehyde for 24 h, followed by 20% sucrose/paraformaldehyde buffer and 30% sucrose/PBS buffer for 72 h. Serial sections (20 µm thickness) were prepared by a freezing microtome. The slices at specific levels were selected for immunohistochemical staining. First, the selected slices were incubated with primary antibodies at 4°C overnight. The primary antibodies included goat anti-4-HNE (Millipore), rabbit anti-GFAP (ProteinTech), cleaved caspase 3 (Cell Signaling Technology), and rabbit anti-NeuN (Millipore). Second, after three washes in 0.3% PBST, slices were incubated with a secondary antibody labeled with a fluorescent dye (Jackson ImmunoResearch Laboratories) for 1 h at room temperature. The slices were then washed and mounted with DAPI Fluoromount-G (Southern Biotech). Finally, images were acquired under a confocal microscope (Nikon), and immunopositive cell quantification in the cortex or hippocampus was performed by ImageJ software.

### Western Blot

The basal cortex and hippocampus of the ipsilateral hemisphere were harvested 72 h after SAH, frozen in dry ice, and stored at -80°C until use. The samples were homogenized and centrifuged for 10 min (12,000 r/min). Equal amounts of protein samples were loaded and probed with antibodies recognizing cleaved caspase 3, 4-HNE (Millipore), cleaved caspase 3 (Cell Signaling Technology), or *β*-actin (Sigma, United States). After incubating with secondary antibodies for 1 h, the membranes were detected by a chemiluminescence substrate and visualized by using a ChemiDoc MP Imaging System (Bio–Rad). The optical density of the bands was measured by ImageJ and normalized to the corresponding *β*-actin band.

### MDA and Total Antioxidant Capability (TAC)

The basal cortex and hippocampus of the ipsilateral hemisphere were homogenized in cold PBS (W/V = 20 mg/100 µL) and then subjected to centrifugal separation. The supernatant was collected for MDA (Beyotime, China) or TAC (Beyotime, China) detection. The detailed testing procedures were carried out according to the manufacturer’s instructions.

### Statistical Analysis

All results are presented as means ± SEM (standard error of the mean). Differences among the four groups were analyzed using one-way or two-way ANOVA using Bonferroni’s multiple comparisons test in GraphPad Prism software 7.0. In all results, a *p* value <0.05 was considered statistically significant.

## Results

Edaravone dexborneol treatment confers long-term protection against neurological dysfunction after SAH.

Male SD rats were randomly assigned to experimental groups and then subjected to SAH or sham surgery. Edaravone dexborneol, edaravone, or the same volume of PBS was administered by tail vein injection twice a day from 1 h to 3 d post-SAH surgery (Figure 1A). The experimental SAH model was induced by an intraluminal puncture of the left middle cerebral artery (MCA). Subarachnoid blood clots were observed on the circle of Willis (Figure 1B). There were no significant differences between the PBS, edaravone, and edaravone dexborneol groups, suggesting that variation in SAH size did not confound the results (Figure 1C).

**FIGURE 2:**
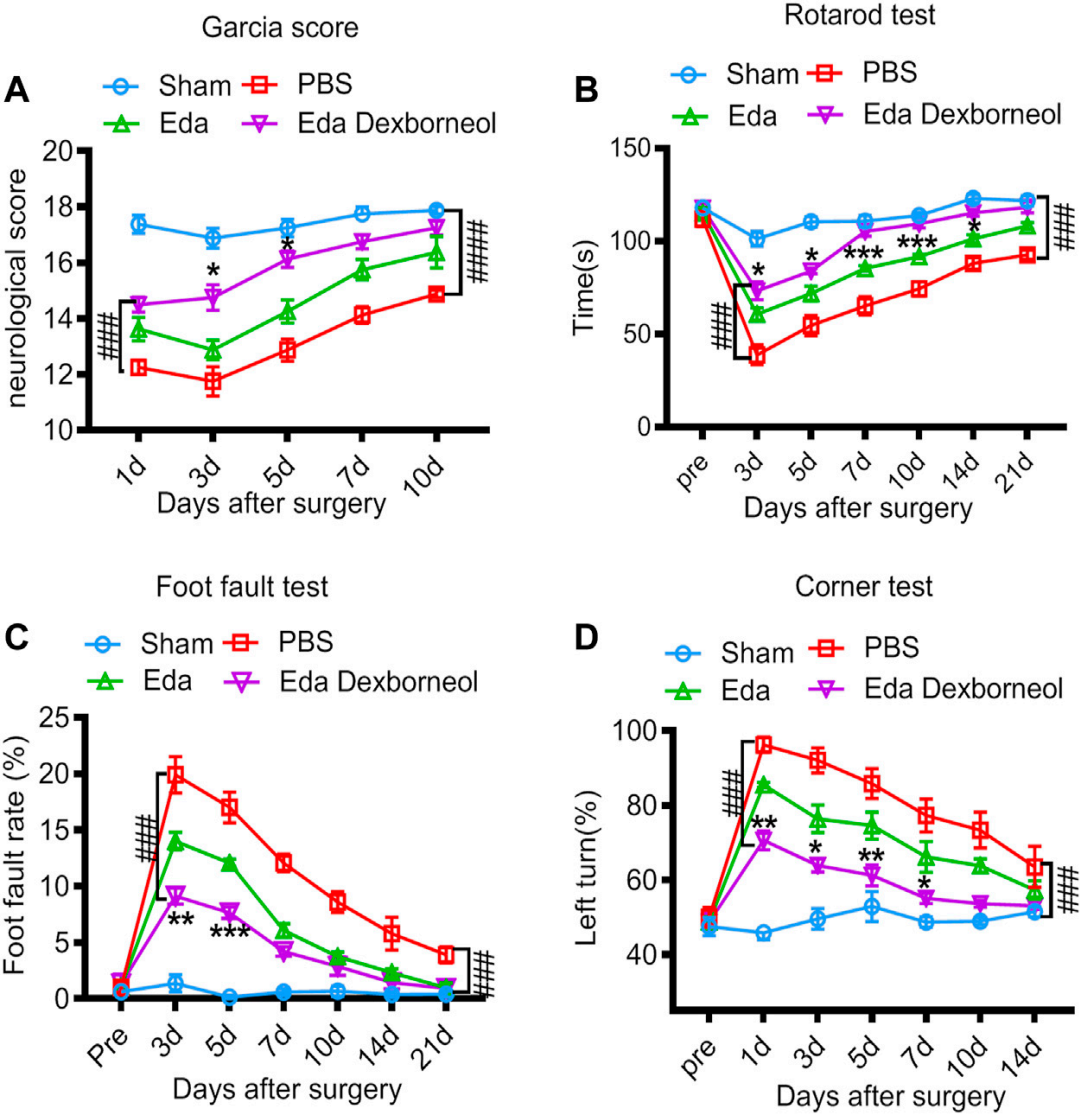
Edaravone dexborneol treatment improves sensorimotor functions after SAH. **(A)** Neurological scores were determined 1–10 days after SAH with the modified Garcia scoring system. **(B–D)** Acute sensorimotor dysfunction at 1–21 days after SAH was assessed by **(A)** Garcia score, **(B)** rotarod test, **(C)** foot-fault test, and **(D)** corner test. N = 8/group. Data are given as mean ± SE. *p < 0.05, **p < 0.01, ***p < 0.001 vs. Eda. ^###^ *p* < 0.001, ^####^ *p* < 0.0001 vs. PBS. Eda: edaravone; Eda dexborneol: edaravone dexborneol.

To assess the therapeutic effects of edaravone dexborneol or edaravone on SAH-induced neurological dysfunction, we employed the Garcia scoring system, rotarod test, foot-fault test, and corner test to examine neurobehavioral capacity before and up to 21 days after SAH. The neurological score results suggested that edaravone dexborneol treatment significantly improved neurobehavioral deficits within 10 days after SAH surgery, and the efficacy was much better than that of edaravone alone at 3 and 5 d post-SAH (Figure 2A). Similarly, sensorimotor deficits were significantly reduced in edaravone dexborneol-treated SAH rats compared to SAH rats treated with edaravone alone, as demonstrated by the performance on the rotarod test for sensorimotor coordination, the foot-fault test for balance function, and the corner test for turning asymmetry (Figures 2B–D). Taken together, these results suggest a better beneficial role of edaravone dexborneol than edaravone alone in improving long-term neurobehavioral functions following SAH.

### Edaravone Dexborneol Treatment Attenuates Neuronal Apoptosis After Subarachnoid Hemorrhage in Rats

**FIGURE 3:**
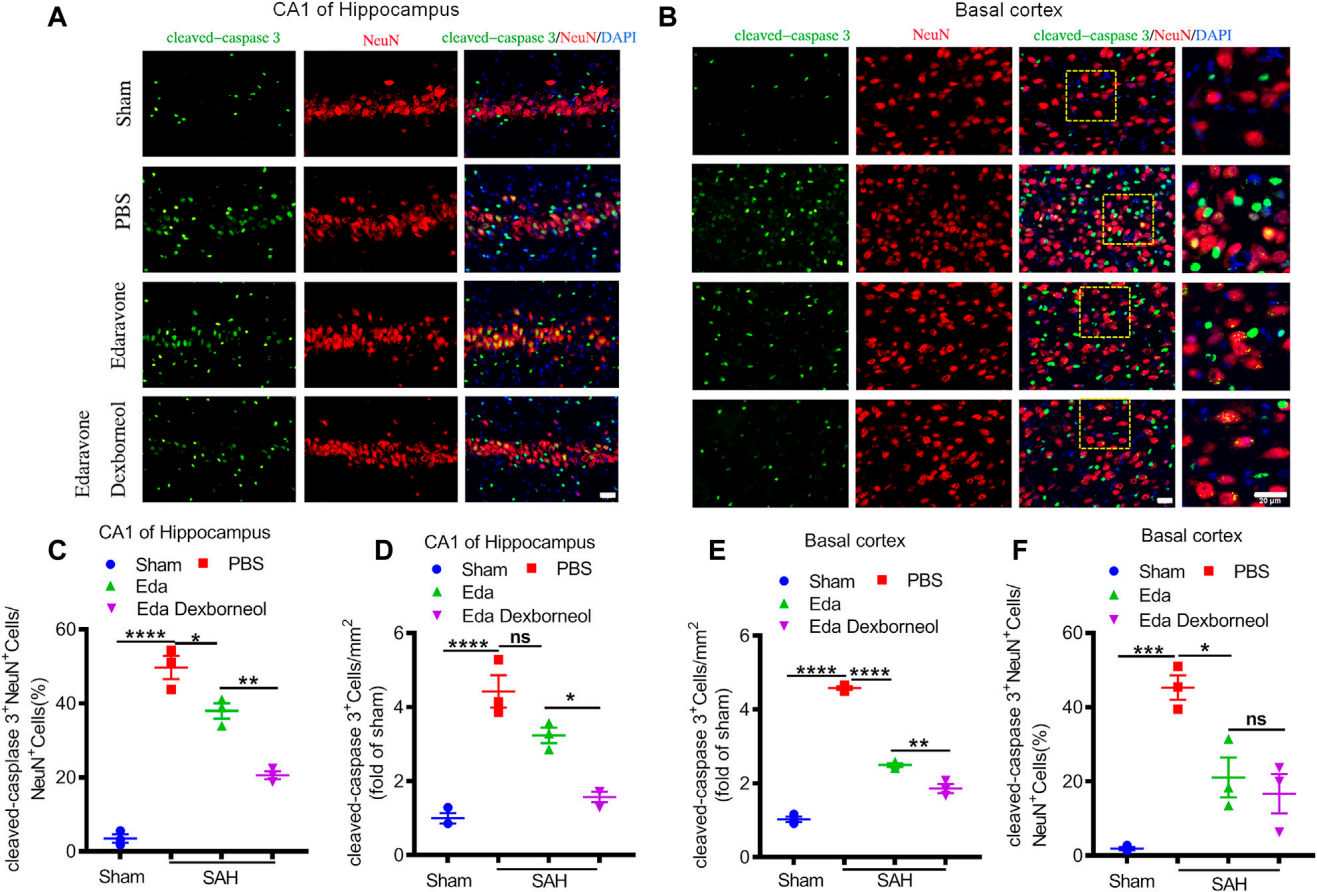
Immunofluorescent staining showing that edaravone dexborneol treatment profoundly decreased the levels of cleaved caspase 3 in the brain after SAH injury. **(A,B)** Representative immunofluorescence images of cleaved caspase 3 (green)/NeuN (red)-stained neurons in CA1 of the hippocampus **(A)** and basal cortex **(B)** at 3 d after SAH. Scale bar: 20 µm. **(C–F)** Quantitative analysis of caspase 3^+^NeuN^+^ neurons and cleaved caspase 3^+^ cells in each group. Data are normalized to sham. *n* = 3/group. Data are given as mean ± SE. *p < 0.05, **p < 0.01, ***p < 0.001, ****p < 0.0001. Eda: edaravone; Eda dexborneol: edaravone dexborneol.

Since neuronal apoptosis has been considered a significant structural basis of neurobehavioral deficits after SAH, we killed the rats 3 days after surgery and detected the expression of cleaved caspase 3 in the CA1 hippocampus and basal cortex by immunofluorescent staining, where cleaved caspase 3 is a key factor leading to apoptosis. As shown in Figures 3A, B, significant increases in cleaved caspase 3 staining were observed in neurons of the CA1 hippocampus and basal cortex regions in PBS-treated rats, resulting in an increased cleaved caspase 3^+^ neuron ratio and cleaved caspase 3^+^ cell number (Figures 3C–F). Both treatments of edaravone dexborneol and edaravone alone significantly decreased the cleaved caspase 3-positive neuron ratio compared to PBS treatment in both brain regions, and treatment with edaravone dexborneol showed greater effects than treatment with edaravone in the CA1 hippocampal region (Figures 3C, E). In addition, edaravone dexborneol treatment was more efficient in decreasing the number of cleaved caspase 3-positive cells in both brain regions (Figures 3D, F).

**FIGURE 4:**
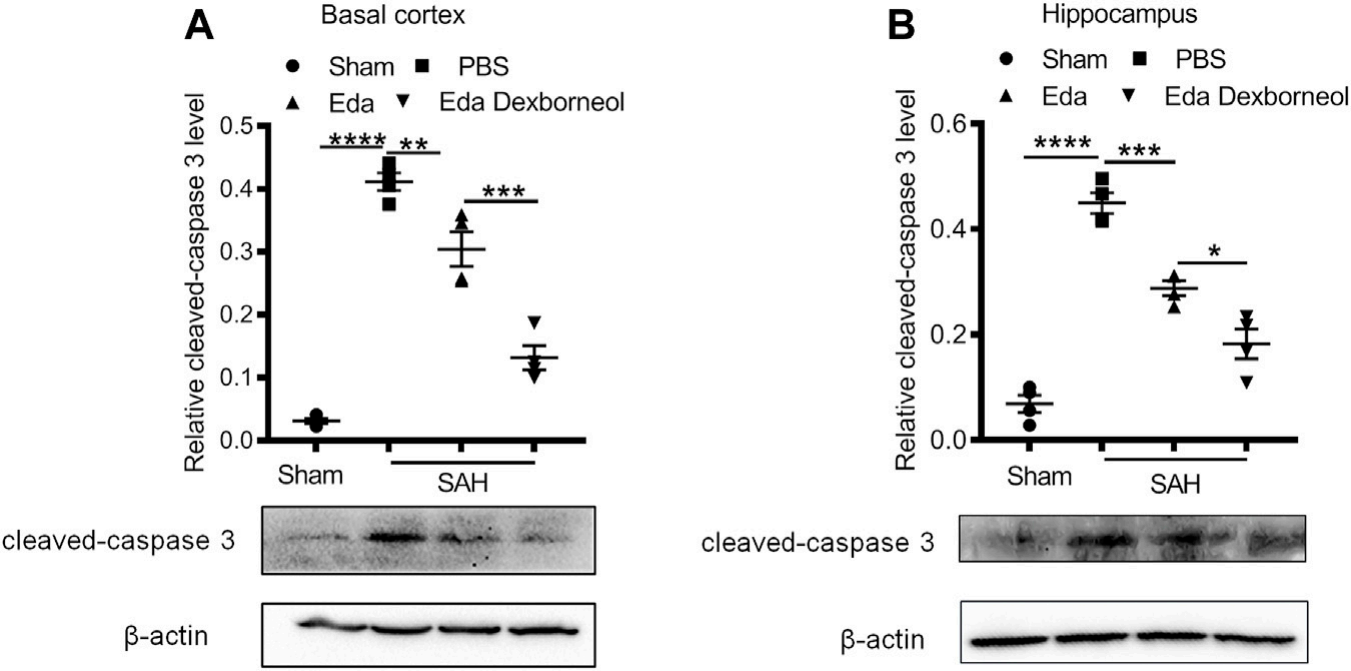
Western blot showing that edaravone dexborneol treatment profoundly decreased the levels of cleaved caspase 3 in the brain after SAH injury. **(A,B)** Representative Western blot images and semiquantitative data show the expression of cleaved caspase 3 in the basal cortex **(A)** and CA1 hippocampus **(B)** at 3 d after SAH. Data are normalized to sham. *n* = 4/group. *p < 0.05, **p < 0.01, ***p < 0.001, ****p < 0.0001. Eda: edaravone; Eda dexborneol: edaravone dexborneol.

Furthermore, to determine the neuroprotective effect of edaravone dexborneol on cerebral cell resistance, we detected the expression of cleaved caspase 3 in the brain basal cortex and CA1 hippocampal regions by Western blot analysis. Western blots validated the immunostaining data (Figures 4A, B). The levels of cleaved caspase 3 increased in both regions 3 days after SAH, while both edaravone dexborneol and edaravone treatments evidently inhibited the expression of cleaved caspase 3. Moreover, edaravone dexborneol showed a better inhibitory effect on cleaved caspase 3. Collectively, these data indicated that compared to edaravone, edaravone dexborneol treatment conferred greater neuroprotection against SAH-induced cell apoptosis, which may underlie the improved long-term neurobehavioral functions.

### Edaravone Dexborneol Treatment Inhibits Oxidative Stress After SAH

**FIGURE 5:**
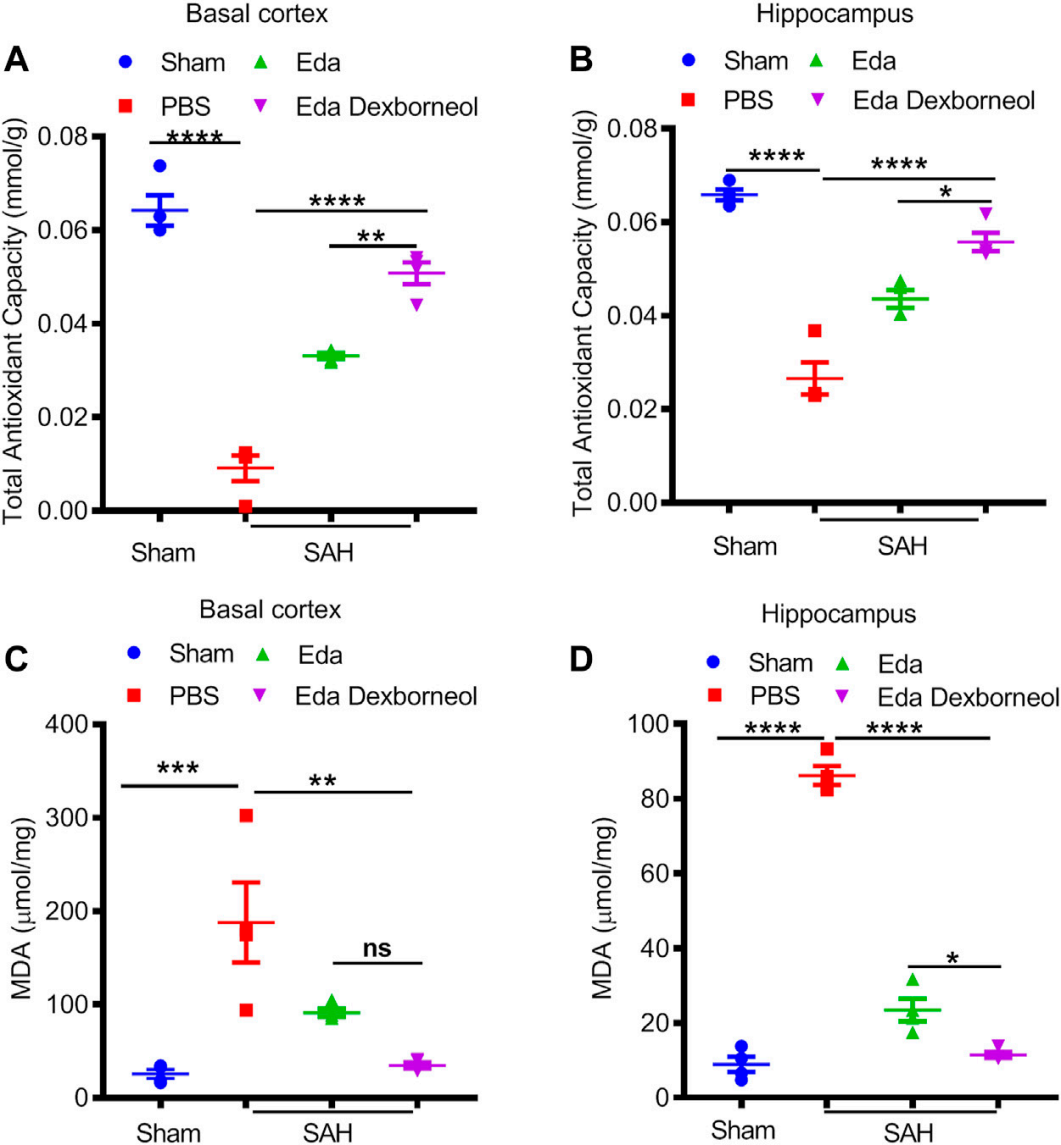
Edaravone dexborneol treatment significantly suppressed oxidative stress after SAH. ABTS assay was used to detect the total antioxidant capability of cortical **(A)** and hippocampal tissues **(B)** after SAH. An MDA assay was used to evaluate SAH-induced lipid peroxidation in the cortex **(C)** and hippocampus **(D)**. Data are given as mean ± SEM. *p < 0.05, **p < 0.01, ***p < 0.001, ****p < 0.0001. Eda: edaravone; Eda dexborneol: edaravone dexborneol.

We next investigated the neuroprotective mechanism of edaravone dexborneol treatment. Oxidative stress is widely considered to be an important mechanism of SAH-induced cell apoptosis, and edaravone has been reported to be a powerful antioxidant. Therefore, we focused on the antioxidant roles of edaravone dexborneol and edaravone treatment, measured by total antioxidant capability (TAC) and lipid peroxidation MDA assays 3 days after SAH. As shown in Figures 5A, B, both edaravone dexborneol and edaravone treatment displayed significant antioxidant functions, and the antioxidant ability of edaravone dexborneol was much stronger than that of edaravone alone. The expression of lipid peroxidation MDA was significantly increased both in the CA1 hippocampus and basal cortex regions 3 d after SAH surgery, which could be inhibited by edaravone dexborneol or edaravone treatment alone (Figures 5C, D). Significantly, compared to edaravone, edaravone dexborneol treatment showed a stronger ability to suppress MDA production in the CA1 hippocampus (Figure 5D). Taken together, these data suggest that edaravone dexborneol treatment confers antioxidant capability against SAH-induced cerebral injury.

Edaravone dexborneol treatment decreases the levels of 4-HNE in the brain after SAH.

**FIGURE 6:**
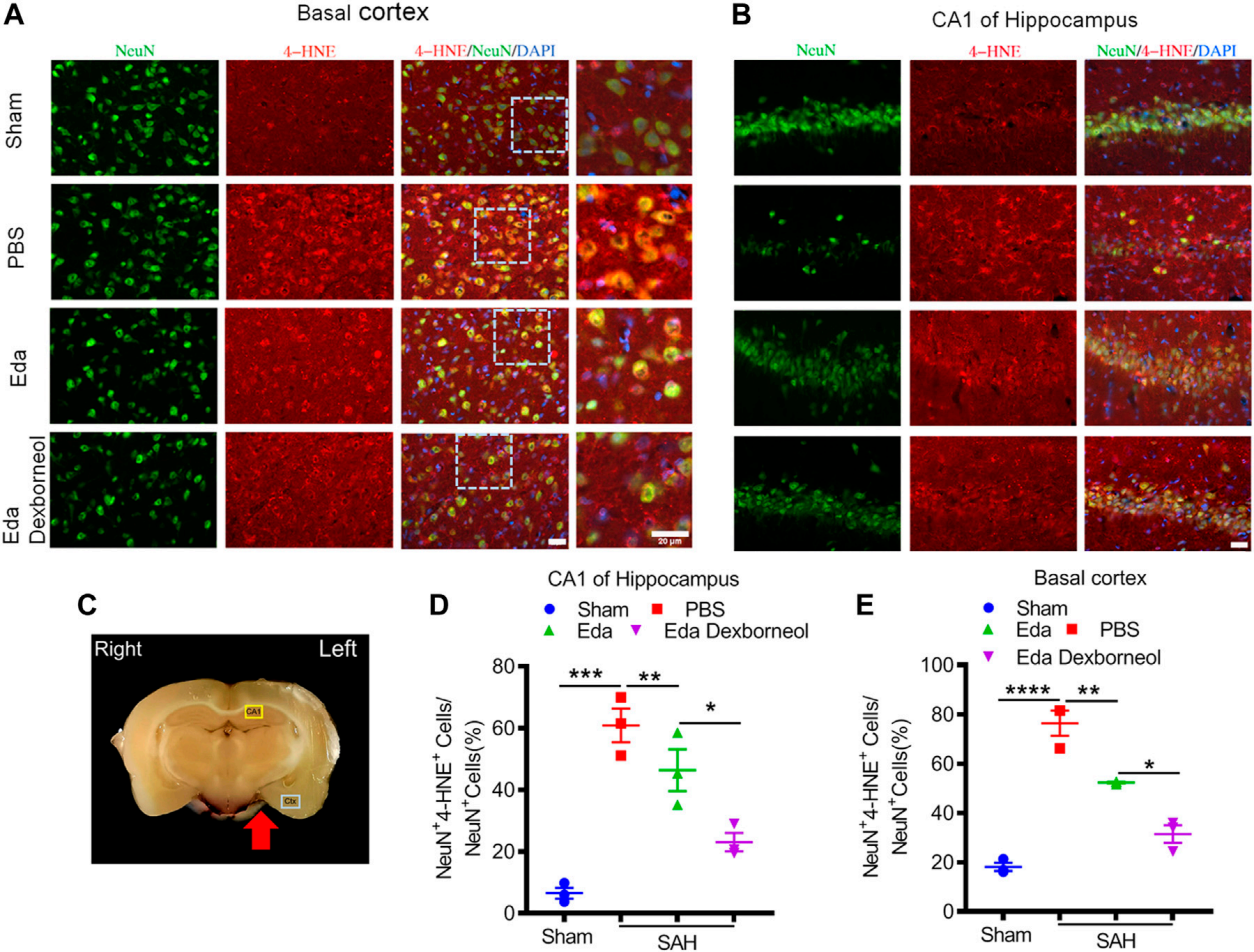
Edaravone dexborneol treatment decreases the levels of 4-HNE in neurons after SAH injury. **(A,B)** Representative immunofluorescence images of 4-HNE (red)/NeuN (green)-stained neurons in the basal cortex **(A)** and CA1 of the hippocampus **(B)** at 3 d after SAH. Scale bar: 20 µm. **(C)** The schema illustrates the regions of interest related to fluorescence staining in the brain cortex or hippocampus. **(D,E)** Quantitative analysis of 4-HNE + neurons in the basal cortex **(D)** and CA1 of the hippocampus **(E)** at 3 d after SAH. *n* = 3/group. Data are given as mean ± SE. *p < 0.05, **p < 0.01, ***p < 0.001, ****p < 0.0001. Eda: edaravone; Eda dexborneol: edaravone dexborneol.

**FIGURE 7:**
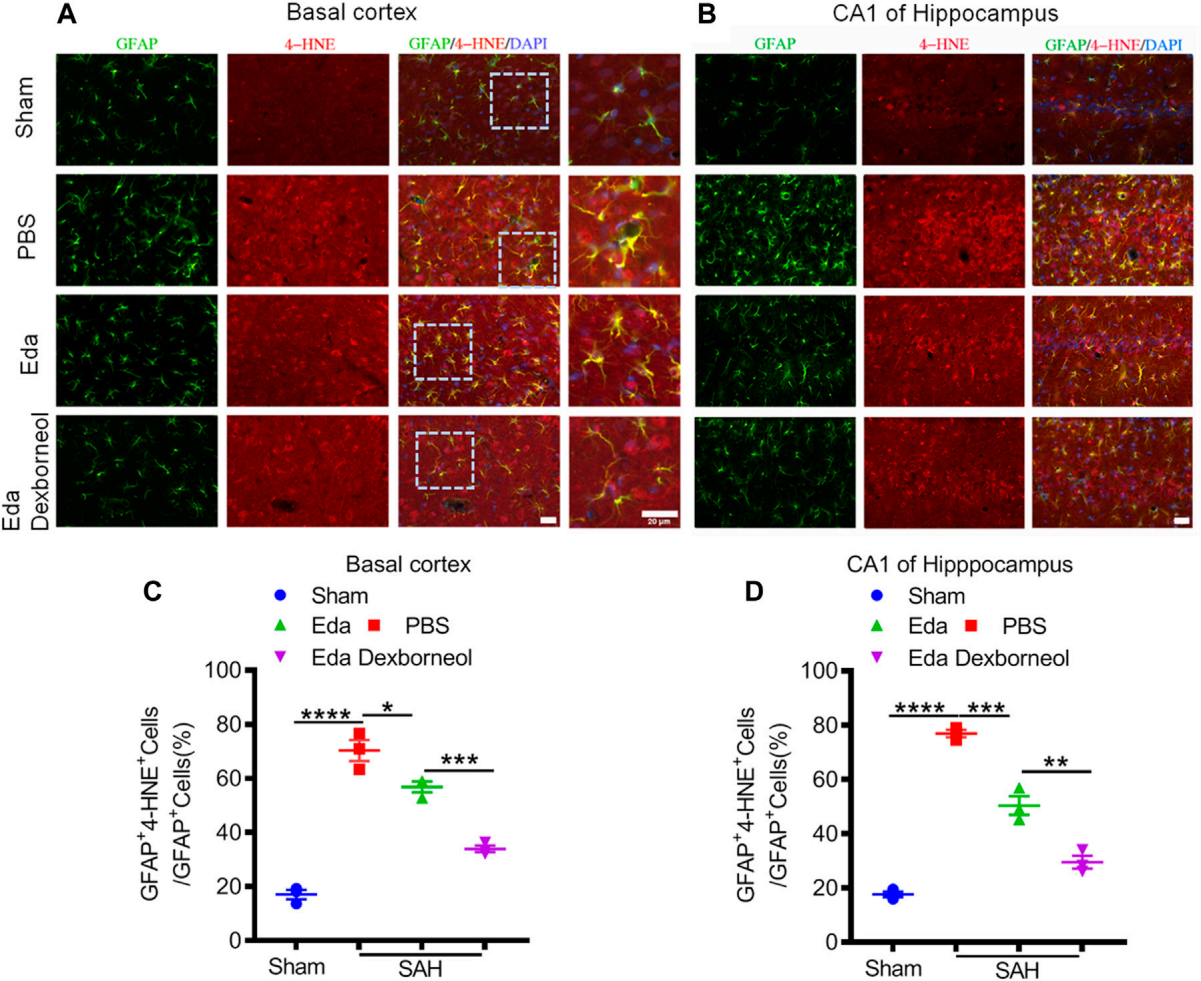
Edaravone dexborneol treatment decreases the levels of 4-HNE in astrocytes after SAH injury. **(A,B)** Representative immunofluorescence images of 4-HNE (red)/GFAP (green)-stained astrocytes in the basal cortex **(A)** and CA1 of the hippocampus **(B)** at 3 d after SAH. Scale bar: 20 µm. **(C,D)** Quantitative analysis of 4-HNE + astrocytes in the basal cortex **(C)** and CA1 of the hippocampus **(D)** at 3 d after SAH. *n* = 3/group. Data are given as mean ± SE. *p < 0.05, **p < 0.01, ***p < 0.001, ****p < 0.0001. Eda: edaravone; Eda dexborneol: edaravone dexborneol.

As an important product of lipid peroxidation, MDA appears to be the most mutagenic product, whereas 4-HNE is the most toxic (Ayala et al., 2014), causing neuronal oxidative damage and death. Next, we investigated the inhibitory effects of edaravone dexborneol on 4-HNE in the brain after SAH. As shown in Figure 6, the 4-HNE level in the neurons were significantly increased both in the basal cortex and CA1 hippocampal regions 3 d after SAH surgery compared with the sham-operated group, whereas edaravone dexborneol or edaravone treatment effectively inhibited the SAH-elevated level of 4-HNE in the neurons, and edaravone dexborneol showed a stronger inhibitory effect. Meanwhile, we detected the level of 4-HNE in astrocytes after SAH using double immunofluorescence staining for 4-HNE and GFAP (Figure 7). Similarly, the results indicated that the 4-HNE level in astrocytes was also significantly increased both in the basal cortex and CA1 hippocampal regions 3 d after SAH surgery. Compared with edaravone treatment, edaravone dexborneol treatment provided a stronger inhibitory effect on 4-HNE levels in astrocytes after SAH.

**FIGURE 8:**
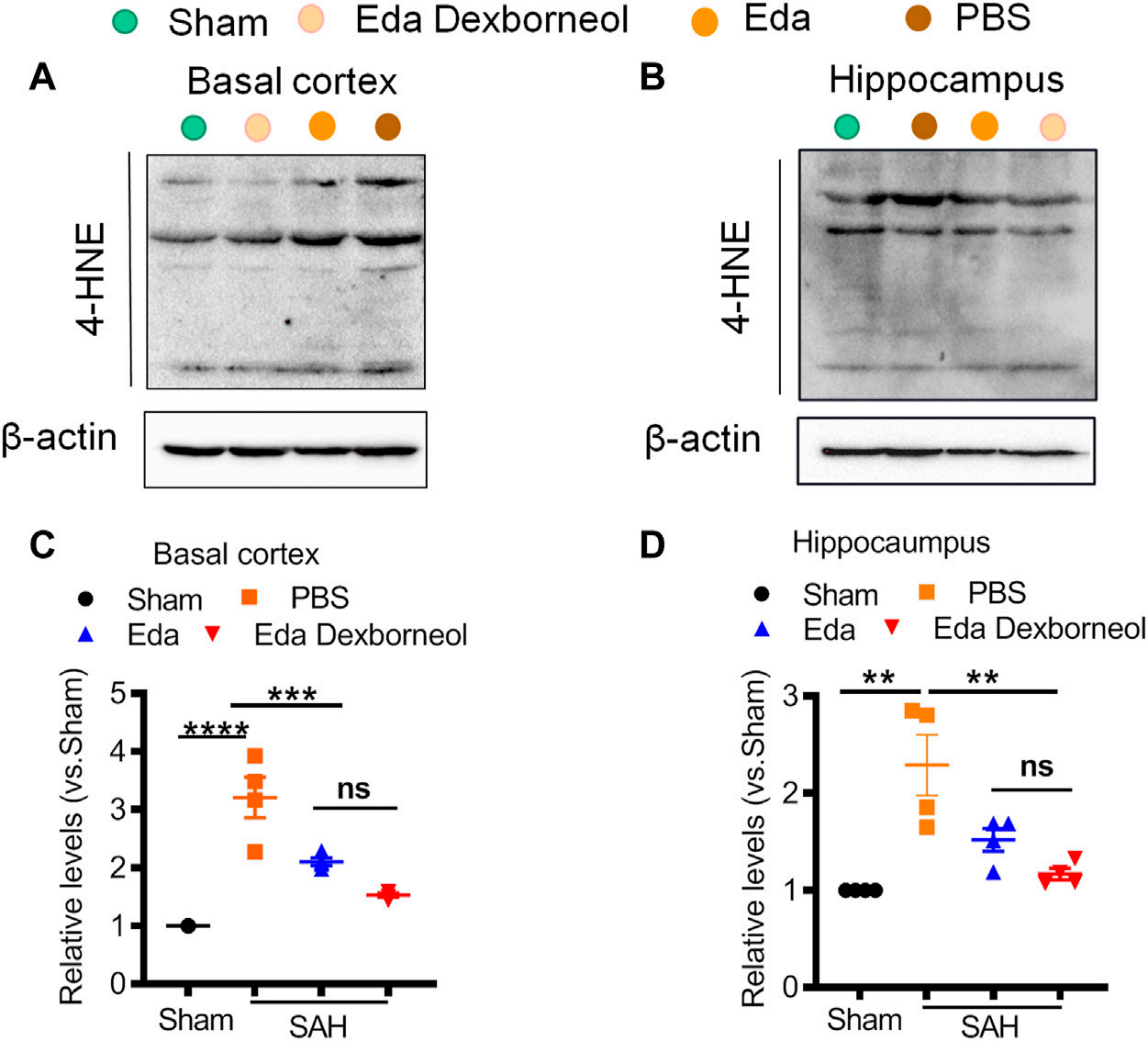
Western blot showing that edaravone dexborneol treatment profoundly decreased the levels of 4-HNE in the brain after SAH injury. **(A,B)** Representative Western blot images show the expression of 4-HNE in the basal cortex **(A)** and CA1 hippocampus **(B)** tissue at 3 d after SAH. **(C,D)** Semiquantitative data of Western blot bands for 4-HNE in each group. Data are normalized to sham. *n* = 4/group. *p < 0.05, ***p < 0.001, ****p < 0.0001. Eda: edaravone; Eda dexborneol: edaravone dexborneol.

Finally, we collected fresh basal cortex and hippocampal tissues 3 d after surgery for Western blotting (Figure 8). The expression of 4-HNE was significantly increased in both the cortex and hippocampus after SAH and could be inhibited by edaravone dexborneol or edaravone treatment. Moreover, Western blot results validated the immunofluorescent staining data showing that the inhibitory effect of edaravone dexborneol on 4-HNE was greater than that of edaravone. In summary, compared with edaravone, edaravone dexborneol treatment was able to provide greater antioxidant ability against SAH-induced brain injury by inhibiting 4-HNE expression.

## Discussion

In this study, we demonstrated that edaravone dexborneol treatment significantly attenuated neuronal apoptosis and improved long-term neurological outcomes up to 21 days after SAH injury. Moreover, we further illustrated that edaravone dexborneol treatment provided a protective effect against SAH injury by, at least partially, inhibiting the 4-HNE-related oxidative stress response.

Edaravone dexborneol is a novel neuroprotective drug that comprises edaravone and (+)-borneol in a 4:1 ratio (Wu et al., 2014b). Phase II and III studies have demonstrated that Chinese patients treated with edaravone dexborneol within 48 h of acute ischemic stroke onset have better functional outcomes than those treated with edaravone alone. Moreover, edaravone dexborneol has been found to be safe and well tolerated at different doses compared with edaravone alone (Xu et al., 2019; Xu et al., 2021). Edaravone dexborneol has been cleared by the National Medical Products Administration (NMPA) of China for clinical use in AIS patients since July 2020 and has obtained good outcomes. The neuroprotective mechanisms of edaravone dexborneol against AIS are thought to involve a multifunctional cytoprotective pathway, including oxidative, excitotoxic, inflammatory, and apoptotic insults. However, the effects of edaravone dexborneol in the treatment of SAH have not been studied.

As a free radical scavenger, edaravone (3-methyl-1-phenyl-2-pyrazolin-5-one) has been widely used to improve functional outcomes in AIS patients in recent years. Furthermore, previous reports showed that edaravone is a useful agent for the treatment of SAH, manifested by a lower incidence of delayed ischemic neurological deficits and a lower incidence of poor outcome in SAH patients (Munakata et al., 2009; Munakata et al., 2011). In order to investigate the advantage of edaravone dexborneol on SAH injury, we chose edaravone as the positive control drug. (+)-Borneol is a bicyclic terpene compound that can prevent nerve injury in ischemic stroke. The related mechanisms of neuroprotection include resistance to reactive oxygen species injury, improvement of cerebral blood flow, blocking of Ca^2+^ overload, and inhibition of neuronal excitotoxicity (Li et al., 2021). Edaravone dexborneol, comprising edaravone and (+)-borneol, is regarded to confer neuroprotection upon stroke injury, which may be more effective than edaravone alone against stroke (Wu et al., 2014b; Xu et al., 2019; Xu et al., 2021). This study is aimed to ascertain the effects of edaravone dexborneol on SAH injury and explore the therapeutic differences between the two drugs. We found that compared to edaravone alone, post-SAH edaravone dexborneol treatment conferred a stronger long-term protection against neurological dysfunction.

Oxidative stress has been identified as an important mechanism by which acute brain damage causes neurological dysfunction after SAH (Jarocka-Karpowicz et al., 2020; Wei et al., 2020). In addition, neuronal apoptosis has been shown to occur in the brain cortex and hippocampus which are associated with oxidative stress injury following SAH (Ayer and Zhang, 2008). Furthermore, the basal cortex and CA1 hippocampus are the most concerned regions that show more severe damage (Sehba et al., 2012; Mo et al., 2019; Zhang et al., 2020). Interestingly, our study showed that compared to edaravone, edaravone dexborneol treatment conferred greater neuroprotection against SAH-induced cell apoptosis in the basal cortex and CA1 hippocampal regions. Under physiological conditions, redox homeostasis is the result of a balance between the expression of oxidative stress products and the antioxidant capacity. Oxidative stress injury occurs when the production of free radical moieties and lipid peroxides exceeds the antioxidant capacity of the related brain regions (Fumoto et al., 2019; Jarocka-Karpowicz et al., 2020). Oxidative stress plays an important role in the course of various pathological changes in early brain injury after SAH, whereas pharmacological or genetic suppression of oxidative stress has been regarded to alleviate early brain injury which is involved in the development of cerebral ischemia and poor outcome (Fumoto et al., 2019). Our study shows that both edaravone dexborneol and edaravone treatment display powerful antioxidant functions according to a total antioxidant capability assay with a rapid ABTS method, and the antioxidant ability of edaravone dexborneol is much stronger than that of edaravone alone. In addition, compared to edaravone, edaravone dexborneol treatment shows a stronger ability to suppress the production of MDA, which may be related to DNA damage after SAH as an important product of lipid peroxide (Zhuang et al., 2019; Jarocka-Karpowicz et al., 2020).

4-HNE is considered as another important lipid peroxidation product not only as a biomarker of oxidative stress but also as one of the most formidable reactive aldehydes (Breitzig et al., 2016; Deng et al., 2020). 4-HNE has been implicated in neuronal oxidative damage and death after SAH because of its toxicity (Siuta et al., 2013). Additionally, recent studies have reported that the cytotoxicity of 4-HNE involves several mechanisms, specifically 1) 4-HNE has been shown to play a role in altering signal transduction by altering protein structure, such as to upregulating proapoptotic factors or accelerating protein degradation (Moldogazieva et al., 2019), and 2) 4-HNE has been reported to promote inflammation by stimulating MCP-1, TNF-α, TGF-β, or other pro-inflammatory cytokines (Vistoli et al., 2013; Zhang et al., 2016). Furthermore, studies report that 4-HNE could induce deactivation of proteins, such as ALDH2, by modes of inhibition or structural change (Jinsmaa et al., 2009). Our data showed that the expression of 4-HNE significantly increased in the brain cortex and hippocampal regions on day 3 after SAH, and edaravone dexborneol treatment significantly suppressed the increase in 4-HNE expression. Thus, the antioxidant ability of edaravone dexborneol is well confirmed. Nevertheless, to verify the key role of 4-HNE in the neuroprotective mechanisms of edaravone dexborneol, we will explore whether supplementation with 4-HNE in the brain could deplete the neuroprotection of edaravone dexborneol in the protection against SAH-induced brain injury. Moreover, future studies are warranted to elucidate the precise signaling mechanism by which edaravone dexborneol inhibits 4-HNE expression after SAH.

Our current study first investigated the therapeutic effects of edaravone dexborneol on an experimental SAH model. Similar to edaravone dexborneol studies on AIS (Wu et al., 2014b), our study further confirmed its antioxidant and anti-apoptosis ability against brain injury. These findings are also consistent with accumulating evidence showing the neuroprotective mechanism of edaravone involving its antioxidant ability (Wu et al., 2014a; Fumoto et al., 2019; Nakanishi et al., 2019). Meanwhile, this was the first study to discuss the inhibitory effect of edaravone dexborneol on MDA and 4-HNE. Similarly, edaravone-alone treatment also showed the inhibition of 4-HNE against traumatic brain injury (Itoh et al., 2010) or AIS (Lukic-Panin et al., 2010)-induced oxidative stress. Furthermore, the effects of edaravone dexborneol on some other mechanisms of SAH-induced brain injury such as neuroinflammation, excitotoxicity, or vessel spasm warrant further investigation. Accompanied by the wide clinical application of edaravone dexborneol on AIS, we look forward to clinical trials on SAH patients.

**FIGURE 9:**
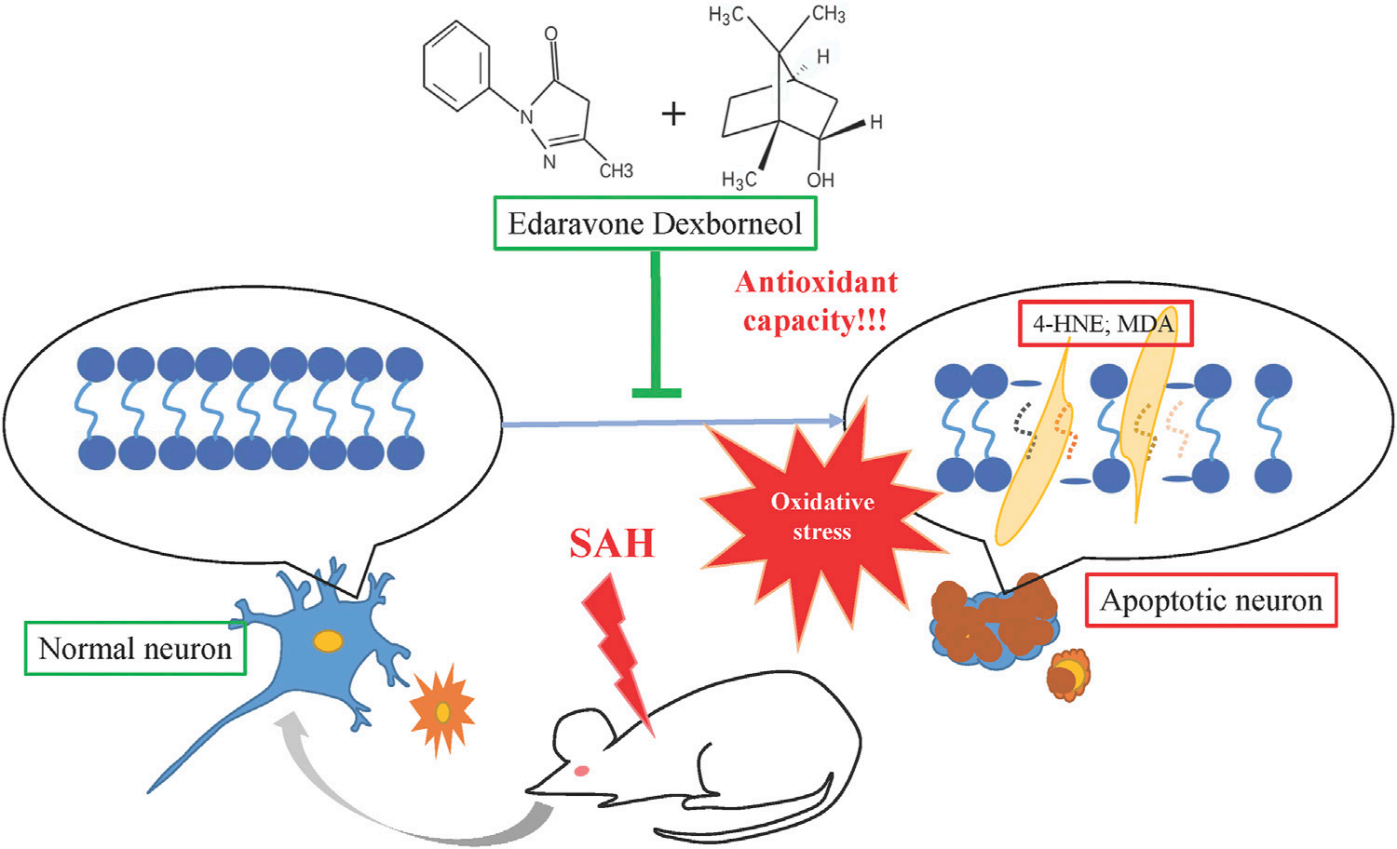
Schematic illustration of the mechanisms by which edaravone dexborneol afforded neuroprotection against SAH-induced brain injury. SAH could cause neuronal apoptosis by inducing oxidative stress injury. Edaravone dexborneol treatment attenuates neuronal apoptosis by affording powerful antioxidant capacity after SAH, accompanied by the inhibition of lipid peroxidation products such as 4-HNE and MDA.

In summary, this study is the first to demonstrate that edaravone dexborneol confers neuroprotection and stabilizes long-term behavioral ability after SAH injury, possibly by improving antioxidant capacity and suppressing lipid peroxidation products such as MDA and 4-HNE, as shown in Figure 9. Our results reveal a novel neuroprotective agent against SAH injury. These results may help develop new clinical strategies for SAH treatment.

## Data Availability

The original contributions presented in the study are included in the article/Supplementary Material, further inquiries can be directed to the corresponding authors.
