# Corrigendum: Edaravone dexborneol treatment attenuates neuronal apoptosis and improves neurological function by suppressing 4-HNE-associated oxidative stress after subarachnoid hemorrhage

**DOI:** 10.3389/fphar.2024.1508703

**Published:** 2024-12-11

**Authors:** Qian Chen, Yichen Cai, Xiaoyu Zhu, Jing Wang, Feng Gao, Mingfeng Yang, Leilei Mao, Zongyong Zhang, Baoliang Sun

**Affiliations:** The Second Affiliated Hospital, Brain Science Institute, School of Basic Medical Sciences of Shandong First Medical University and Shandong Academy of Medical Sciences, Taian, China

**Keywords:** oxidative stress, subarachnoid hemorrhage, edaravone dexborneol, 4-HNE, edaravone

In the published article, there was an error in **Supplementary Image 1**. The inclusion of multiple brain images in the figure led to a typesetting oversight, resulting in the duplication of two brain images within the PBS and Eda Dexborneol groups. The correct **Supplementary Image 1** is now published with the published article.

The authors apologize for this error and state that this does not change the scientific conclusions of the article in any way. The original article has been updated.

