# Supplementary figures and images for: Edaravone Dexborneol Treatment Attenuates Neuronal Apoptosis and Improves Neurological Function by Suppressing 4-HNE-Associated Oxidative Stress After Subarachnoid Hemorrhage
Source: Front Pharmacol. 2022 Apr 21;13:848529. doi: 10.3389/fphar.2022.848529 (PMC9068884; doi:10.3389/fphar.2022.848529)

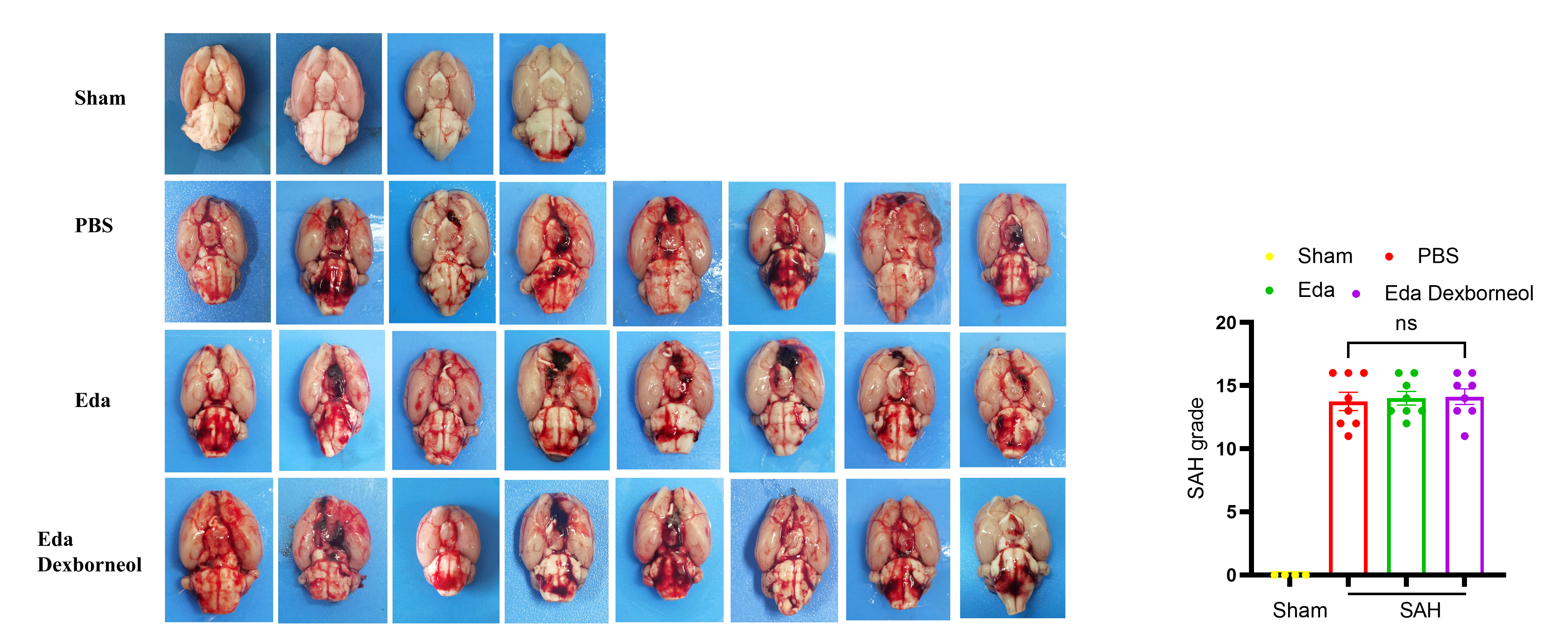

Supplement: Supplementary file 1 [file Image1.TIF]
